# Label-Free Electrochemical Microfluidic Chip for the Antimicrobial Susceptibility Testing

**DOI:** 10.3390/antibiotics9060348

**Published:** 2020-06-20

**Authors:** Hyoil Jeon, Zeeshan A. Khan, Emad Barakat, Seungkyung Park

**Affiliations:** School of Mechanical Engineering, Korea University of Technology and Education, Cheonan, Chungnam 31253, Korea; wjsgydlf@koreatech.ac.kr (H.J.); acezeeshan@live.com (Z.A.K.); elemad1987@gmail.com (E.B.)

**Keywords:** AST, microfluid chip, UTIs, label-free, *E. coli*

## Abstract

The emergence and spread of antibiotic-resistant bacteria is a global threat to human health. An accurate antibiotic susceptibility test (AST) before initiating the treatment is paramount in the treatment and bacterial resistance control. However, the current AST methods either are complex, use chemical and biological labels, lack multiplexing, are expensive, or are too slow to be used for routine screening. The primary objective of the current study is to develop an automated electrochemical microfluidic chip (EMC) for simple and rapid AST. The microfluidic channels and gold microelectrodes were designed for the automation of antibiotic mixing and distribution in multiple test chambers and for electrical signal measurements. The designed chip was tested for AST with *E. coli* samples, and the results were compared with conventional broth microdilution. The presented EMC provided rapid bacterial count and AST in 170 and 150 min, respectively, while the conventional broth microdilution evaluates in 450 and 240 min, respectively. The rapid AST capability of the EMC was further demonstrated with the artificial urine samples, and the results were obtained in 270 min, which was 90 min faster than the broth microdilution method. Additionally, the minimum inhibitory concentration (MIC) was evaluated on the EMC and compared with the results from an AlamarBlue assay. The experimental results indicate the sensitivity of the chip, minimum loss of antibiotics, and eventually, reduction in the evolution of antibiotic resistance. Cumulatively, we have developed an automated, label-free, economical, rapid, robust, and user-friendly EMC for the evaluation of AST in urine samples.

## 1. Introduction

Antibiotics have saved millions of lives. However, owing to the capability of bacteria to grow in the presence of all the current antibiotics including gentamicin, ciprofloxacin, and sulfamethoxazole/trimethoprim, the antibiotic resistance in bacteria has become a global threat [1,2,3,4]. Currently, antimicrobial resistance (AMR) in bacteria accounts for more than 700,000 deaths per year worldwide. Besides, it is predicted to increase by 700%, i.e., approximately 10 million deaths and a loss of US$ 100 trillion by the year 2050 [5]. The most common bacterial infection is Urinary Tract Infections (UTIs), and the development of AMR has become a significant challenge in treating UTIs [6]. Several cross-sectional studies have demonstrated substantial increases in bacterial resistance, for example, in 2017 in England, 34% of analyzed UTI samples were reported to be resistant to various antibiotics as opposed to 29.1% in 2015 [7,8]. Several studies from China, the Indian subcontinent, and Africa have postulated a convergence that a minimum of 65% of *E. coli* isolated from urine specimens of UTI is antibiotic resistant [1,9,10,11]. It is speculated that AMR can increase the rate of UTI associated mortality by 9% [12]. In addition, the development of novel antibiotics has declined in the 21st century and fewer than 10 drugs were developed in the first decade of 2000 as compared to 1990–2000 when 22 drugs were developed [13,14]. Cumulatively, the rapid increase in UTIs with AMR and the decline in novel drugs pose a significant challenge towards the treatment of infections.

Researchers are regularly reporting new resistance mechanisms which include structural or functional modifications in the antibiotic binding sites, development of spatial protection of the drug targets, and production of metabolites which can directly modify or inactivate the antibiotics [4]. The initiation and progression of resistance mechanisms are promoted by the indiscriminate use of antibiotics, self-medication, household storage, and limited diagnostic equipment leading to misdiagnosis, improper dosage, and injudicious prescription [15,16,17,18]. The improper selection of the drug to which a pathogen has a low susceptibility can lead to unnecessary and extended exposure to the antimicrobial; to prolonged ailment and hospitalization; subsequently, to increases the cost of medication; to futile dedication of skillful manpower; to disease progression; and finally, to acquisition of resistance in bacteria [19,20]. Owing to the higher incidences of antibiotic-related malpractices and to the lack of economical tools that offer a rapid diagnosis of antibiotic resistance, the progression rate of antibiotic-resistant microbes is higher in developing countries. Thus, the development of an accurate and inexpensive device for antibiotic susceptibility testing (AST) is essential not only for the patient’s health but also to repress the rapid expansion of antimicrobial resistance.

AST provides crucial information on the selection as well as minimum inhibitory concentration (MIC) evaluation of antibiotics to be used in treatment against bacterial infections [21]. Disk diffusion is considered as the gold standard, but it can get influenced by physiochemical factors like solubility, pH, and temperature, and the most significant disadvantage is the sluggish nature of the process as it takes more than 16 h [22,23]. Broth macrodilution is another reliable, simple, cost-effective, and common method for determination of susceptibility. The key weakness associated with the macrodilution method was the requirement of a large quantity of chemicals that can be overcome by microdilution. However, lack of automation for preparing serial dilutions, long incubation time of more than 12 h, and chances of cross-contamination are still major challenges associated with microdilution [24,25]. Automated susceptibility tests such as MicroScan WalkAway and Vitek provide a considerable advantage over manual methods by providing simplified workflow and quantitative results [26,27]. Despite their benefits, these automated systems are still dependent on bacterial growth and turbidity changes; hence, the analysis remains slow and requires approximately 4.5–18 h [26,27]. The bulky and expensive nature of these automated instruments is another factor that limits their usability [28]. The limitations in the manual and automated systems could be addressed by developing a miniaturized, portable, turbidometry-independent automated system.

A molecular tool such as Polymerase Chain Reaction (PCR) amplifies the resistant gene for AST. Recently, an assay targeting the β-lactamase gene was developed for AST. The PCR-based assays are fast and sensitive. However, several reports have described the existence of several isoforms of β-lactamases, and spontaneous mutations in the target genes add to the complications of PCR as it is impractical to design a universal primer for AST evaluation [29]. Matrix-assisted laser desorption ionization-time of flight (MALDI-TOF) offers ultra-sensitivity by specific detection of antibiotic resistance proteins [30]. Nonetheless, false-positive results in AST arise owing to the interaction of small-sized antibiotics (approximately 1 kDa) with the matrix used for preparing samples for MALDI-TOF [31]. The large size of instruments and prohibitive cost of equipment, consumables, and maintenance further limit the AST analysis with MALDI-TOF [28,31]. Alternatively, colorimetric and fluorescent biosensors, labeled with reporter proteins and enzymes such as green fluorescent protein and horseradish peroxidase; dyes like SYTOX; and pH indicators have been developed to reduce the size and cost of the instrument and the required sample volume and to provide rapidity with accuracy to AST [28,32]. Despite all the benefits, colorimetric and fluorescent biosensors suffer as the output signal collection is dependent on the external optics such as microscope or spectrophotometer. Moreover, as the labeled proteins and some dyes are temperature sensitive, the storage of such biosensors requires low-temperature refrigeration. Improper storage can lead to degradation of proteins or enzyme activity, leading to a false negative or high background signal [33]. Consequently, a fallible antibiogram often compels the physicians to prescribe broad-spectrum antibiotics, thereby intensifying the problem of antibiotic resistance. Therefore, the development of a simple automated, non-labeled, reliable, and portable antibiotic susceptibility device is a pressing need.

Micro-total analysis systems (μTAS) can be a smart candidate for susceptibility testing if we can develop an appropriate strategy by the implication of microfluidics. Along with miniaturization of the device, a microfluidic chip offers a large surface-to-volume ratio, so it can provide a suitable environment for the rapid multiplication of bacteria [34,35,36]. Recently, Baltekin et al. has developed a phase-contrast-based microfluidic chip capable of determining AST in UTI samples [37]. Samples were injected in a polydimethylsiloxane (PDMS) chip which has two rows of cell traps; in each row, there are 2000 cell traps of dimension 1.25 × 1.25 × 50 μm that had undergone proliferation for 2 h. A single bacterium gets trapped in the microchannels containing antibiotics, resistant strain proliferates. The single-cell proliferation inside the test chambers was examined continuously through time-lapse phase-contrast microscopy, leading to AST within 30 min of sample loading [37]. However, to determine the MIC, repeated experiments with different concentrations of antibiotics are needed. Moreover, the chip has limited application due to its complex microfabrication process and the requirement of time-lapse phase-contrast microscopy. Furthermore, due to the relatively small channel size, the device can suffer from channel fouling and clogging [38,39]. An electrical impedance-based chip with a built-in heater that allows susceptibility evaluation within 90 min has also been developed [40]. However, the chip requires a complicated process including several injections for the introduction of bacteria, antibiotics, and antibodies on the microchannels, followed by multiple washing steps, which restrict frequent use and commercialization of the device. Another study has demonstrated the use of a electrochemical biosensor for sensitive AST based on the detection of precursor rRNA [41]. In similar research, an electrochemical sensor platform with an AC electrokinetic process was developed for AST and detection of the bacterial species based on 16s rRNA [42]. Both the assays offer sensitivity, specificity, and rapidity but lack automation in antibiotic dilution and distribution, which increases the chance of cross-contamination and error in antibiotic dilution, leading to false MIC values. However, those assays highlight the benefits of implementing electrochemical sensing for AST.

In this paper, we have designed and proposed an electrochemical microfluidic chip (EMC) for simple and rapid AST with the automation of processes. The microfluidic channels and microelectrodes are designed for the automation of antibiotic mixing and distribution in multiple test chambers and for electrical signal measurements. Operational simplicity and efficiency based on the on-chip, label-free detections are demonstrated with the experimental study of bacterial growth and AST in tryptic soy broth (TSB) media and artificial urine media (AUM) samples. The test results have then been compared with the broth microdilution method to validate the rapidity and accuracy of the EMC.

## 2. Results and Discussion

### 2.1. Bacterial Growth Analysis and MIC

**Figure 1:**
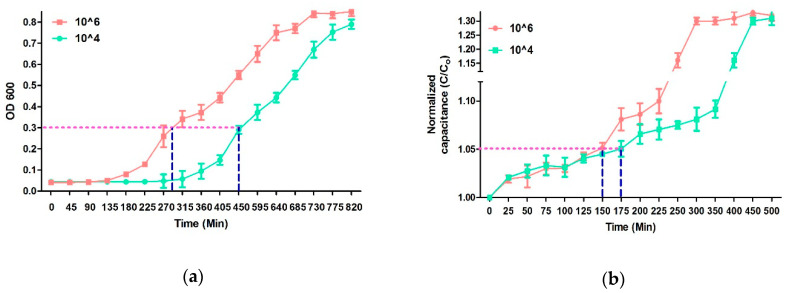
The graphs represent the bacterial growth kinetic analysis of 10^6^ and 10^4^ CFU/mL *E. coli* by (**a**) the change in optical density at 600 nm with time and (**b**) the change in normalized capacitance with time.

Figure 1 shows the change in standard capacitance following *E. coli* proliferation. The proliferation of bacteria utilizes the organic nutrients present in the media through metabolism and produces charged ions and polar molecules; thus, the generated cations such as K^+^ and Na^+^ are exchanged through bacterial ion channels [43]. Theoretically, the double layer capacitance ($C_{dl}$) is summarized by the following equations [44]:

(1) $$C_{dl}=\frac{\varepsilon_{dl}A}{d},\;and\;\varepsilon_{dl}=\varepsilon_{0}\varepsilon_{\rho },$$

where $\varepsilon_{dl}$ is the permittivity of the bilayer, $\varepsilon_{0}$ is the permittivity of the free space, $\varepsilon_{\rho }$ is the dielectric constant, d is the thickness of the double layer, and A is the area of the electrode. Because of the increase in double layer capacitance with bacterial growth, the concentration of the bacterial solution can generally be characterized by the change in absolute capacitance (Figure S1).

Sezonove et al. have demonstrated that, owing to the depletion of utilizable carbon sources, the steady-state proliferation of *E. coli* ceases around 0.3 optical density at 600nm (OD 600 nm) [45]. The doubling time during steady-state growth of *E. coli* is approximately 20 min. By plotting mass/cells vs OD 600, Sezonove et al. have shown a significant change in the curve at OD 0.3. This change in the curve at OD 600 was due to the sudden decrease in average cell mass, which is indicative of a decreasing growth rate and clearly marks the end of steady-state growth [45]. Therefore, this point was considered as the cutoff value to compare the cell density dependent growth rate of *E. Coli*. Moreover, the time required to reach OD 0.3 is dependent upon the initial density of the bacterial cells. The initial density of the bacteria in the solution can be observed by the comparative analysis of the growth curve. Previous studies have shown that a change of 5% in impedances in the growing culture indicates a significant change in the initial density-based bacterial growth [46]. Similarly, we have utilized the normalized capacitance to generate the bacterial growth curve. A comparative analysis of growth curve obtained from the optical density measurement up to 0.3 (OD 600 nm) and 5% difference in the normalized capacitance revealed that developed microfluidic chip can indicate a difference between 10^6^ and 10^4^ *E. coli* in 175 min while the conventional microplate reader requires 450 min (Figure 1). Initially, the OD 600 and normalized capacitance values were the same for both 10^4^ and 10^6^ samples; however, after proximately 130 min, the curves start to deviate from each other. The 10^6^ *E. coli* curve starts to move quickly at the Y-axis while 10^4^ samples moves slowly in the same axis. Optimal growth rates were observed, during which cell count doubled at discrete time intervals. Then, the cell division and death of bacteria counterbalances, resulting in no net increase in cell numbers and no net change in OD or capacitance [46,47]. The reduction in growth rate is usually due to a lack of nutrients and/or a buildup of toxicity due to the presence of debris of dead bacteria in the solution, indicating the stationary phase (Figure 1). Furthermore, it should be noted that, to determine a significant change in the initial density of cells, the cutoff for OD 600 measurement should reach 0.3. On the other hand, only a 5% increase in the normalized capacitance indicates a significant change in the initial bacterial density. Therefore, it can be concluded that this inherent difference in the cutoff value along with rapid growth due to a large surface-to-volume ratio are primarily responsible for the variation in the estimation time of the initial density of bacteria between OD and normalized capacitance [34,35,36].

**Figure 2:**
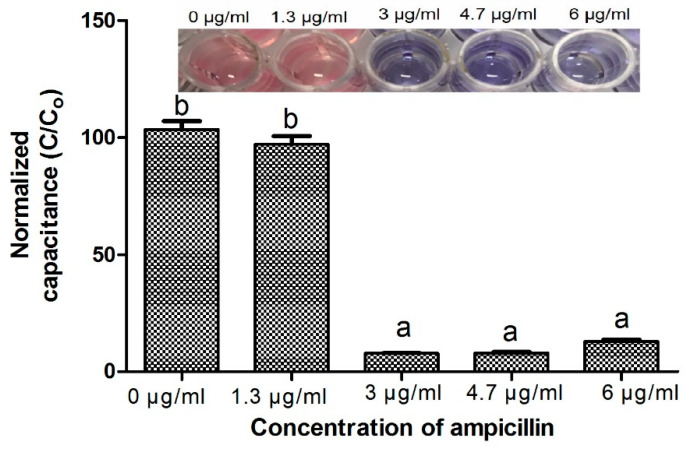
The graph shows an antibiotic susceptibility test (AST) by an AlamarBlue assay using different concentrations of ampicillin. Error bars represent the standard deviation from the mean values of three experiments (different letters indicate different levels of significance: * *p* < 0.05).

When the bacterial suspension treated with various concentrations from 0 μg/mL and 1.3 μg/mL of ampicillin were tested with the AlamarBlue, a uniform blue color was observed in the initial phase. After 4 h of incubation, the blue color changed to pink due to the reduction of resazurin (the active compound in AlamarBlue) to resafurin by growing bacteria in 0 and 1.3 μg/mL ampicillin samples. However, in the cases of the 3.0, 4.7, and 6.0 μg/mL antibiotic samples, after the addition of AlamarBlue, the color of the solution remained blue after 4 h of incubation (Figure 2). The changes in color are calculated in terms of percentage reduction of AlamarBlue. The one-way ANOVA showed that percentage reduction is statistically significant between the 0–1.3 µg/mL and 3.0–6.0 µg/mL concentrations. Therefore, it can be concluded that the MIC of ampicillin was approximately 3.0 µg/mL. No statistically significant difference was observed in percentage reduction of alamar blue above MIC (Figure 2). These results were used to set the concentration gradient of ampicillin for further research to avoid the loss of antibiotics and to make the whole process cost-effective.

### 2.2. Analysis of Microplate and Microfluidic Chip-Based AST in TSB

**Figure 3:**
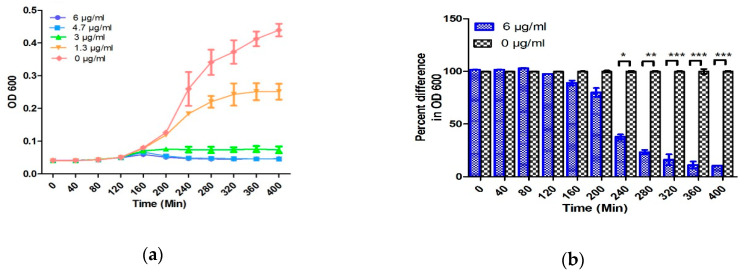

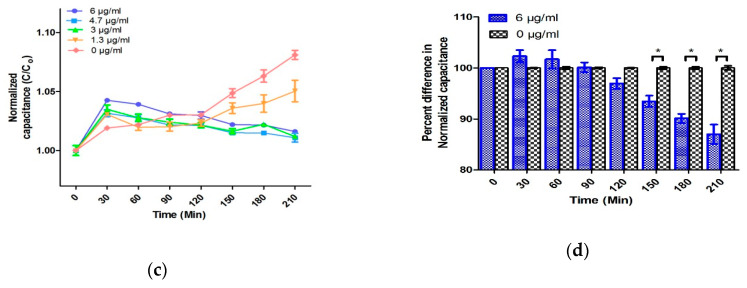
AST evaluation of 10^6^ CFU/mL *E. coli* in TSB media: (**a**) The change in the OD 600 nm with time, (**b**) the percentage change in the OD between control and antibiotic-treated samples, (**c**) the change in normalized capacitance with time; and (**d**) the percentage change in the normalized capacitance between control and antibiotic-treated samples. Error bars represent the standard deviation from the mean values of three experiments (significance indicators: * *p* < 0.05, ** *p* < 0.01, and *** *p* < 0.001).

The performance of the EMC for AST has been evaluated by testing the 10^6^ CFU/mL of *E. coli* in TSB. Figure 3 shows the results of AST of *E. coli* towards ampicillin by broth microdilution and the microfluidic chip.

The growth curve obtained from broth microdilution followed the standard growth curve consisting of an initial lag phase, followed by a log phase, and then the stationary phase. Previous studies have shown the bactericidal activity of a suprainhibitory concentration (<10 µg/mL) of ampicillin on the 10^6^ CFU/mL of *E. coli*, and the difference between the growth curves generated with and without antibiotic can be seen in 240–300 min [48]. Similar results have been obtained in the present study by broth microdilution with 10^6^ CFU/mL of *E. coli* (Figure 3a,b). Change in turbidity was observed in the samples below 3.0 μg/mL which reflects the MIC of ampicillin to be 3 μg/mL.

At the first two concentrations of 0 and 1.3 μg/mL, the normalized capacitance of the sample steadily increases. On the other hand, at concentrations above 3.0 μg/mL of antibiotic, the normalized capacitance of the samples increases for the initial 20–30 min and decreases afterward. The initial enhancement in the C/C_0_ value in the lag phase is because the bacteria tend to acclimatize to the new environment in this phase [38]. Due to acclimatization, the action of antibiotic is lowest at this point and cells are formed with deficient cell walls [38]. Moreover, Fridman et al. have postulated that the variation in the lag time is considered as the first change made by bacteria in response to antibiotic stresses, which indicates the generation of various types of bacteria to facilitate their evolution and therefore the development of antibiotic resistance [47]. The initial change in the normalized capacitance can be speculated due to the slow denaturation of antibiotics in TSB media. To understand this phenomenon, two control experiments including measuring the capacitance of various concentrations of ampicillin (0, 1.3, 3.0, 4.7, and 6.0 µg/mL) in TSB and AUM have been performed. As performed in the absence of *E. coli,* no change in the absolute capacitance was observed over time (Figure S2). Therefore, comparative analysis of samples with and without bacteria may conclude that the change in capacitance is a cumulative effect of antibiotics, media, and bacterial growth. After the initial lag phase, ampicillin strongly prevents the proliferation of bacteria by covalently binding with the enzyme transpeptidase. Transpeptidase is essential for the formation of the cross-linkage between peptidoglycan strands required for cell wall biosynthesis [49]. This halts the biosynthesis of cell walls, thereby showing the bactericidal activity. Owing to the bactericidal activity, the production of polar molecules reduces, which eventually decreases the C/C_0_ [50]. Most researchers have utilized a suprainhibitory concentration of antibiotics to show rapid AST results [40,51]. However, using the suprainhibitory concentration of antibiotics may increase the expense of AST and leads to wastage of the precious antibiotics, which can accelerate the evolution of antibiotic resistance in bacteria [22]. Due to the large surface-to-volume ratio and the gas-permeable PDMS, the rapid growth of bacteria is obtained without external agitation or oxygenation. These characteristics of PDMS-based design facilitates the EMC performance, and AST can be accomplished within 150 min without using suprainhibitory concentrations of antibiotics (Figure 3c,d). A similar AST time (150 min) was obtained even by using MIC of ampicillin in 10^6^ *E. coli* CFU in TSB media (Figure S3). Furthermore, five different concentrations of antibiotic are automatically generated and tested by utilizing only 90 µL of culture and 10 µL of ampicillin. The low volume requirement and high sensitivity towards a narrow range of antibiotics aids in AST as well as accurate MIC determination. Adding to that, it also reduces the wastage of antibiotics, media, and water. The MIC values obtained by AlamarBlue assay, OD 600, and the normalized capacitance measurement operated by the developed EMC were the same value of 3.0 µg/mL. The identical MIC of conventional methods and the EMC demonstrate the reliability and accuracy of the chip.

### 2.3. Antibiotic Susceptibility Testing in Artificial Urine Samples

The performance of the designed EMC has further been validated for bacteria susceptibility testing in an artificial urine sample. Both 10^4^ CFU/mL, the lower range of clinically relevant UTI, and standard 10^6^ CFU/mL of *E. coli* were spiked in the artificial urine, and AST was evaluated by EMC [37].

**Figure 4:**
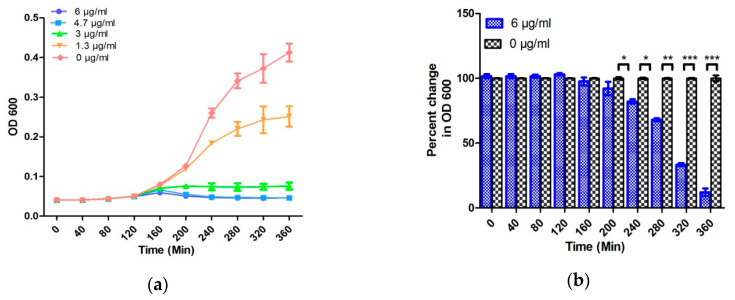

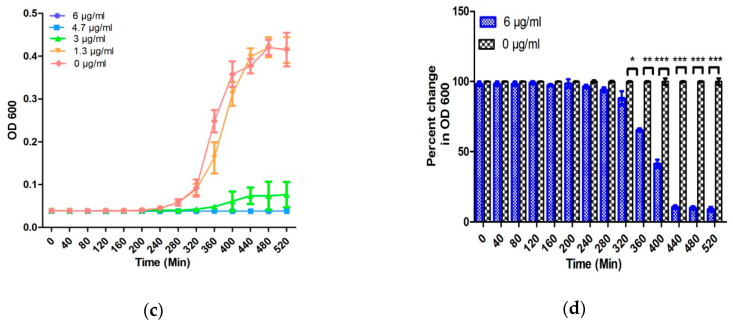
The graphs represent AST evaluation in artificial urine samples: (**a**) the change in OD 600 nm with time in 10^6^ CFU/mL *E. coli* samples, (**b**) the percentage change in the OD 600 nm between control and antibiotic-treated 10^6^ CFU/mL *E. coli* samples, (**c**) the change in the OD 600 nm with time in 10^4^ CFU/mL *E. coli* samples, and (**d**) the percentage change in the OD 600 nm between control and antibiotic-treated 10^4^ CFU/mL *E. coli* samples. Error bars represent the standard deviation from the mean values of three experiments (significance indicators: * *p* < 0.05, ** *p* < 0.01, and *** *p* < 0.001).

**Figure 5:**
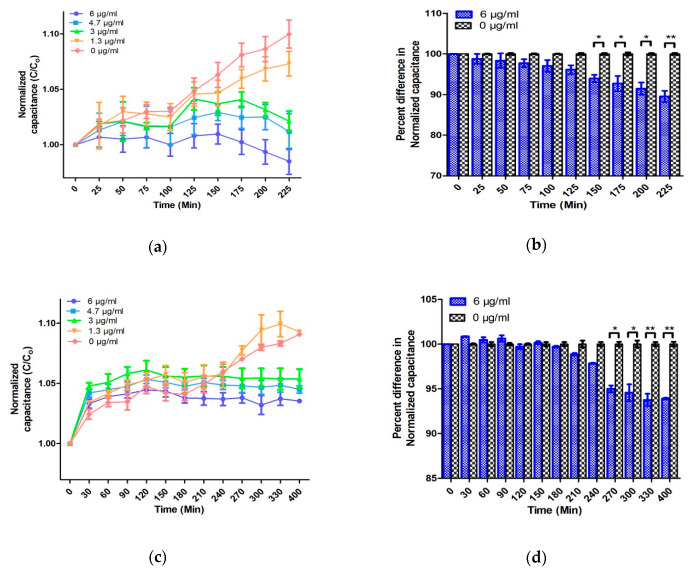
The graphs represent AST in artificial urine samples: (**a**) the change in normalized capacitance with time in 10^6^ CFU/mL *E. coli* samples, (**b**) the percentage change in the normalized capacitance between control and antibiotic-treated 10^6^ CFU/mL *E. coli* samples, (**c**) the change in normalized capacitance with time in 10^4^ CFU/mL *E. coli* samples, and (**d**) the percentage change in the normalized capacitance between control and antibiotic-treated 10^4^ CFU/mL *E. coli* samples. Error bars represent the standard deviation from the mean values of three experiments (significance indicators: * *p* < 0.05, ** *p* < 0.01, and *** *p* < 0.001).

For 10^6^ CFU/mL in TSB and artificial urine, the change in normalized capacitance as well as optical density is comparable and results can be obtained in 240 and 150 min, respectively (Figure 3, Figure 4 and Figure 5). Moreover, the EMC results with 10^4^ CFU/mL can be obtained within 270 min, which is 90 min faster than the conventional broth microdilution method (Figure 4 and Figure 5). However, a significant change in the normalized capacitance was observed at 175 min instead of 150 min when a percentage change was calculated with an MIC value with a 10^6^ sample (Figure S4). Similarly, in case of 10^4^ samples, a statistically significant change in the normalized capacitance was noticed at 300 min instead of 270 min when percentage change was calculated with 3 µg/mL ampicillin (Figure S4). This indicates that EMC results are media dependent when the MIC of ampicillin is used. Nonetheless, these changes in AST evaluation time with different media were not observed with 6 µg/mL ampicillin, reflecting that robust results can be obtained with EMC by using a higher concentration of ampicillin. Furthermore, in the AUM samples, the initial changes in normalized capacitance in response to the ampicillin concentration was found to be in the opposite manner to the TSB culture experiments (Figure 5). To understand this initial difference in normalized capacitance, a comparative analysis of ampicillin in TSB and AUM in the absence (control group) and the presence of *E. coli* was performed. No change in capacitance was observed overtime in the control group, while a considerable change in the normalized capacitance was observed in the samples containing *E. coli* (Figure 3, Figure 5 and Figure S2). Interestingly, a minor change in the absolute capacitance was observed between the control groups, which suggests that the culture conditions are important for the behavior of the capacitance but that the variation in capacitance overtime is solely due to the interaction of antibiotics, media, and bacterial growth. Considering the user-friendly operation, use of low concentration of antibiotics, room-temperature storage of EMC, and non-labeled detection leading to extremely low false results, the speed to obtain the AST result for the clinically relevant sample is respectable.

As *E. coli* is responsible for 85% of all the diagnosed UTIs in primary care, we have spiked the artificial urine sample with this species; however, EMC is not limited to UTI and other species can also be evaluated. Additionally, since the EMC is not color or turbidity dependent, the same principle can be used for other AST determinations in the clinically significant sample such as milk, blood, or cerebrospinal fluid without compromising the sensitivity of the test. The EMC is fabricated from standard photolithography, which can readily provide a batch process for mass production with cleanroom facilities. However, for scaling up the production of the proposed EMC, the cost and speed of fabrication can further be reduced by using the optimized protocol, e.g., large-scale fabrication of 3D microstructures developed by Nguyen et al. [52,53]. Similarly, instead of using a licensed commercial software (LabView), Android-based applications can be developed and installed on cellphones or tablets to provide portability and to reduce the overall cost [54].

## 3. Materials and Methods

### 3.1. Fabrication of the EMC

**Figure 6:**
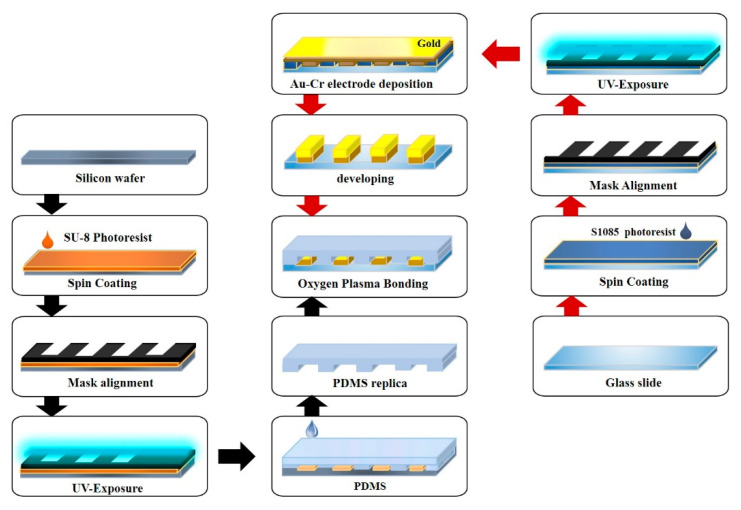
Schematic representation of the electrochemical microfluidic chip (EMC) fabrication processes.

Figure 6 shows a schematic of the EMC fabrication. Standard photolithography was utilized to deposit the gold-chromium microelectrodes on the glass surface. For electrode fabrication, the electrode pattern was made by CAD software (AutoCAD v2018, Autodesk, San Rafael, CA, USA) and printed on a transparency film. A positive photoresist (S1805, Shipley, Marlborough, MA, USA) was poured on the glass slide followed by the spin coating process (Midas systems, Daejeon, South Korea) for a uniform distribution of the photoresist over the glass slide. The coated slides were exposed to UV light in a mask aligner (MDA-400S, Midas, Daejeon, South Korea) to get the desired pattern. The pattern was then developed in the S1805 developer solution to remove the unwanted photoresist. Subsequently, 30-nm gold and 15-nm chromium were deposited by a sputter machine (Q300T D, Quorum, East Sussex, UK) and lifted-off by Ultrasonication (UB-405, powersonic, Seoul, South Korea) in the acetone solution. For fabrication of the channel mold, a procedure similar to electrode fabrication was followed. A negative photoresist (SU-8 2025, Microchem, Newton, MA, USA) was utilized to get the desired pattern on the silicon wafer. After mold fabrication was accomplished, that mold was used to produce any further PDMS channels. For channel fabrication, a PDMS (Dow Corning, Midland, MI, USA) with a 10:1 ratio was poured on the fabricated mold and cured at 65 °C for 2 h. The patterned PDMS was gently peeled off from the mold (the mold should be cleaned and stored for further use), and sample inlet chambers and bacterial chambers were drilled on the prefabricated PDMS with the Biopunch (Healthlink, Jacksonville, FL, USA). The slides with the gold–chromium electrode and the PDMS channel were bonded by oxygen plasma (Vita Plasma, Femtoscience, Gyeonggi, South Korea). The bonded EMC was heated at 60 °C for 2 h for permanent bonding.

### 3.2. Operation of EMC

**Figure 7:**
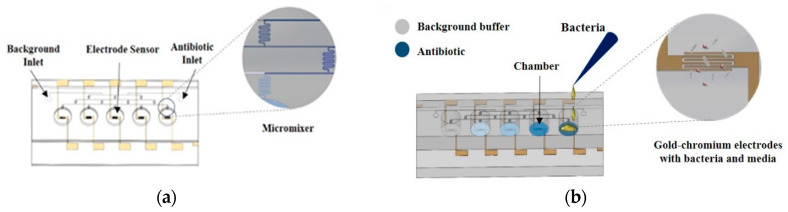
Schematic representation of the proposed electrochemical microfluidic chip showing (**a**) inlet, electrodes and micromixer and (**b**) bacterial and antibiotic loading in the micro well.

Figure 7 represents the components and functioning of EMC for evaluating susceptibility. A 60 µg/mL ampicillin and the background buffer (water) were simultaneously injected at the inlet through a syringe pump (NE-4000, National Direct Network, Bangkok, Thailand) at an operating flow rate of 10 µL/min. According to the designed structure of the tree-shaped micromixer, antibiotics are automatically mixed with the water, diluted, and distributed to the corresponding chambers in five different concentrations, i.e., 0, 13, 3, 47, and 60 µg/mL. Final concentrations of 0, 1.3, 3.0, 4.7, and 6.0 µg/mL ampicillin were developed through the addition of 90 µL of TSB containing 10^6^ CFU/mL *E. coli*.

### 3.3. Bacterial Culture, Growth Analysis, and Antibiotic Susceptibility Testing

The *E. coli* (Seattle 1946 (DSM 1103, NCIB 12210)) culture obtained from the Korean Collection for Type Culture (KCTC) was prepared from single overnight-grown colonies suspended in a test tube containing TSB media (BD Difco, Swindon, Wiltshire, UK) and incubated for 12 h at 37 °C with shaking. For counting colony-forming units (CFUs), the grown culture was plated on Luria Bertani agar (BD Difco, Swindon, Wiltshire, UK & Samchun, Daejeon, South Korea) plates and incubated for overnight at 37 °C, followed by CFU counting. The counted samples were diluted to the desired cell density for further experiments. The bacterial growth curve was generated by continuous monitoring of turbidity with an optical spectrophotometer (SpectraMax, Molecular Devices, San Jose, CA, USA) (optical density (OD) at λ = 600 nm). For minimum inhibitory concentration (MIC) estimation, a single bacteria colony was picked, grown in TSB media, and incubated overnight at 37 °C. The overnight culture was diluted with a ratio of 1:100 in TSB and grown in 96-well plates up to 0.5 McFarland units. Five different concentrations of ampicillin (Sigma-aldrich, St. Louis, MO, USA) viz. 0, 1.3, 3.0, 4.7, and 6.0 µg/mL were added to the bacterial samples. After 24 h of incubation, 10% AlamarBlue (Invitrogen, Waltham, MA, USA) was added to each well and the plates were again incubated for 4 h at 37 °C with gentle shaking. The absorbances were measured at 570 nm and 600 nm by the microplate reader. The percent reduction of AlamarBlue (R) was calculated by following the manufacturer’s instructions with the following equation [55]:

(2) $$R=\frac{\left(\varepsilon_{ox}\lambda_{2}\right)\left(A\lambda_{1}\right)-\left(\varepsilon_{ox}\lambda_{1}\right)\left(A\lambda_{2}\right)}{\left(\varepsilon_{red}\lambda_{1}\right)\left(\overset{´}{A}\lambda_{2}\right)-\left(\varepsilon_{red}\lambda_{2}\right)\left(\overset{´}{A}\lambda_{1}\right)}\times 100,$$

where $\varepsilon_{ox}$ and $\varepsilon_{red}$ are constants representing the molar extinction coefficient of AlamarBlue oxidized form (blue) and the molar extinction coefficient of AlamarBlue reduced form (pink), respectively; A represents the absorbance of test wells, while $\overset{´}{A}$ represents the absorbance of negative control wells (only media plus AlamarBlue); $\lambda_{1}$ = 570 nm; and $\lambda_{2}$ = 600 nm.

The antibiotic susceptibility testing was performed by continuous monitoring of bacterial growth in the presence of five concentrations of antibiotic (0, 1.3, 3.0, 4.7, and 6.0 µg/mL) at OD 600 nm. Control experiments were performed by measuring the absolute capacitance of various concentrations of ampicillin (0, 1.3, 3.0, 4.7, and 6.0 µg/mL) in TSB media for 8 h. All experiments were repeated three times.

### 3.4. Preparation of Artificial Urine Sample

The artificial urine used in the experiments was prepared by following the standard protocol, and the components are listed in Table 1 [56]. All the components were dissolved in ultrapure water (18.2 MΩ-cm) followed by filtration through a membrane filter with a pore size of 0.2 μm. TSB was used to simulate the bacterial growth, and the pH was adjusted to a value of 5.8. Two different concentrations of bacterial samples were suspended in artificial urine at the concentrations of 10^6^ and 10^4^ CFU/mL; 90 µL of the prepared samples was manually pipetted in the microwells containing various concentrations of antibiotic. Control experiments were performed by measuring the capacitance of various concentrations of ampicillin (0, 1.3, 3.0, 4.7, and 6.0 µg/mL) in artificial urine media for 8 h. All experiments were repeated three times.

### 3.5. Electrical Signal Measurements

The EMC was connected with the LCR meter (E4890AL, Keysight, Santa Rosa, CA, USA), which was further connected with a computer to monitor and collect the capacitance signal. The LCR meter was controlled by LabVIEW software (LabVIEW NXG 1.0, National Instruments, Austin, TX, USA). The capacitance was measured at 1 mV voltage, 1 kHz frequency, and intervals of 30 s. After the samples were injected, the microwells were sealed with tape to prevent evaporation. Electrical signals of all the samples were measured at a stable temperature of 37 °C, maintaining the EMC inside the incubator. The capacitance values (C) obtained at various time points after incubation were normalized with the capacitance values of 0-h samples (C_0_), and the results were represented in the form of normalized capacitance (C/C_0_).

### 3.6. Statistical analysis

Percentage change between the control and antibiotic treated groups was compared by two-tailed Student’s *t*-test (significance indicators: * *p* < 0.05, ** *p* < 0.01, and *** *p* < 0.001). Changes in the AlamarBlue reduction were analyzed by one-way ANOVA followed by Tukey’s post hoc test (*p* < 0.05 was considered as statistically significant). Both analyses were performed by SPSS software (SPSS 16.0 software; Macrovision Corporation, Santa Carlo, CA, USA).

## 4. Conclusions

In the present study, we report a label-free, rapid (<150 min) AST using EMC. Real-time comparative analysis of capacitance produced by bacterial growth at various concentrations of antibiotics generated by built-in micromixers has been utilized to achieve AST. The micromixers make the device easy-to-use, as it automatically generates a series of a concentration gradient. The EMC was used to analyze the different bacterial counts by comparing the growth curve and AST determination in standard TSB media. The EMC requires 175 min and 150 min for the bacterial count and AST, respectively, while OD 600 entails 450 min for the bacterial count and 240 min for AST. The results demonstrate the superior speed of EMC compared to the conventional microplate reader. Furthermore, the developed EMC was further tested with spiked artificial urine samples of *E. coli* at a clinically relevant concentration of 10^4^ CFU/mL to mimic the UTI sample. The results were generated within 270 min, which is 90 min quicker than the turbidity measurements. The device does not utilize the commonly used redox molecules like resazurin, ferrocyanide, or any other redox concentration optimization for electrochemical monitoring. Thus, it offers fewer false results and room-temperature storage of chips. The EMC has the potential for AST for different sets of samples as well as various strains of bacteria. The addition of full automation which can facilitate the loading of bacterial samples and a portable capacitance analyzer will greatly enhance the applicability of the developed EMC. AST of the clinical samples is required to establish the competency of the EMC. Moreover, further studies are warranted to fully understand the relationship between capacitance, different types of media including human serum and saliva, bacterial cells, and the various antibiotics.

## Figures and Tables

**Table 1:** Artificial urine composition [56].

Component	Quantity (g/L)
CaCl_2_·2H_2_O	0.651
MgCl_2_·6H_2_O	0.651
NaCl	4.6
Na2SO4	2.3
Na_3_C_6_H_5_O_7_	0.65
Na_2_C_2_O_4_	0.023
KH_2_PO_4_	2.8
KCl	1.6
NH_4_Cl	1.0
CO(NH_2_)_2_	25.0
C_4_H_9_N_3_O_2_	1.1
Tryptic soy broth	10.0

## Supplementary Materials

The following are available online at https://www.mdpi.com/2079-6382/9/6/348/s1, Figure S1: Absolute capacitance (Farads) of various CFU of E. coli., Figure S2: Absolute capacitance (Farads) of various concentrations (0, 1.3, 3.0, 4.7 and 6.0 µg/mL) of ampicillin: (a) TSB media; (b) Artificial urine media (AUM), Figure S3: Graphs representing AST in TSB using 3.0 µg/mL ampicillin, Figure S4: Graphs representing AST in the artificial urine samples using 3.0 µg/mL ampicillin.
