# Supplementary material for: Label-Free Electrochemical Microfluidic Chip for the Antimicrobial Susceptibility Testing
Source: Antibiotics (Basel). 2020 Jun 20;9(6):348. doi: 10.3390/antibiotics9060348 (PMC7344617; doi:10.3390/antibiotics9060348)
Supplement: Supplementary file 1 [file antibiotics-09-00348-s001.pdf]

Supplementary Information

Label-free electrochemical microfluidic chip for the antimicrobial susceptibility testing

Hyoil Jeon [1,†], Zeeshan A. Khan [1,†], Emad Barakat [1] and Seungkyung Park [1,*]

[1] School of Mechanical Engineering, Korea University of Technology and Education, Cheonan, Chungnam 31253, South Korea

* Correspondence: spark@koreatech.ac.kr

† These authors contributed equally.

Received: 18 May 2020; Accepted: date; Published: date

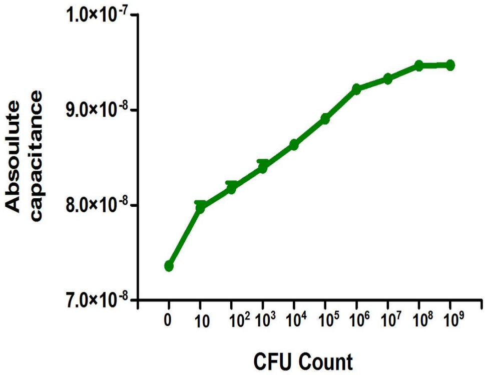

Supplementary Figure 1. Absolute capacitance (Farads) of various CFU of *E. coli*.

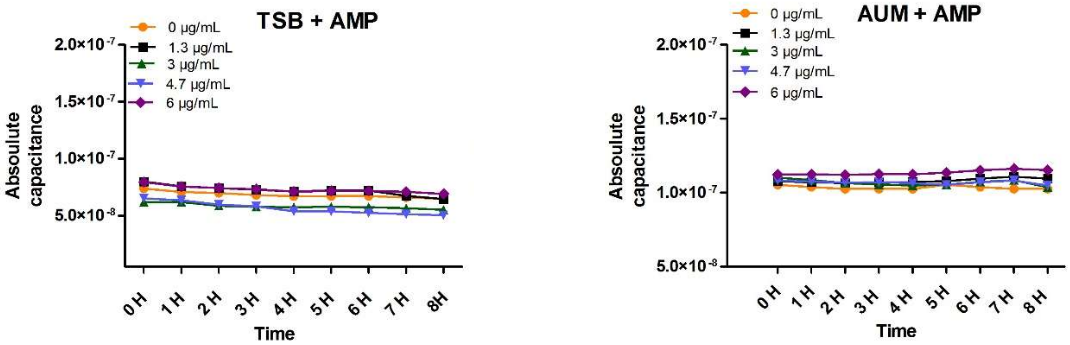

(**a**) (**b**)

Supplementary Figure 2. Absolute capacitance (Farads) of various concentrations (0, 1.3, 3.0, 4.7 and 6.0 μg/mL) of ampicillin: (**a**) TSB media; (**b**) Artificial urine media (AUM).

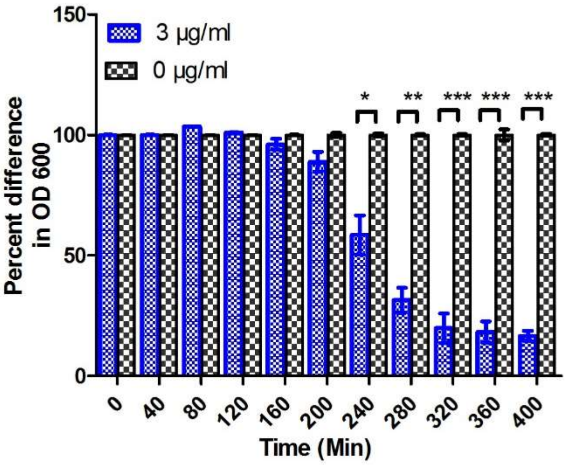

(**a**)

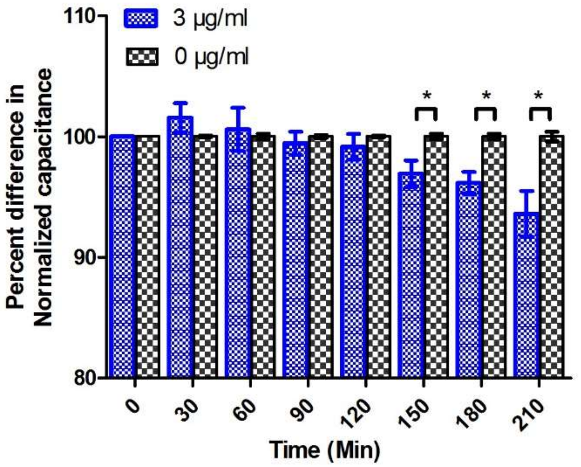

(**b**)

Supplementary Figure 3. Graphs are representing AST in TSB using 3.0 μg/mL ampicillin: (**a**) The percentage change in the OD600 between control and antibiotic-treated 10^6 CFU/mL *E. coli* samples; (**b**) The percentage change in the normalized capacitance between control and antibiotic-treated 10^6 CFU/mL *E. coli* samples. Error bars represent the standard deviation from the mean values of three experiments (significance indicators: *p < 0.05, ** p < 0.01, and *** p < 0.001).

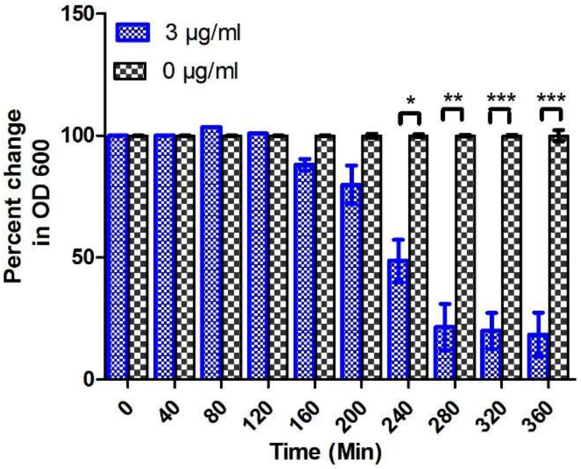

(**a**)

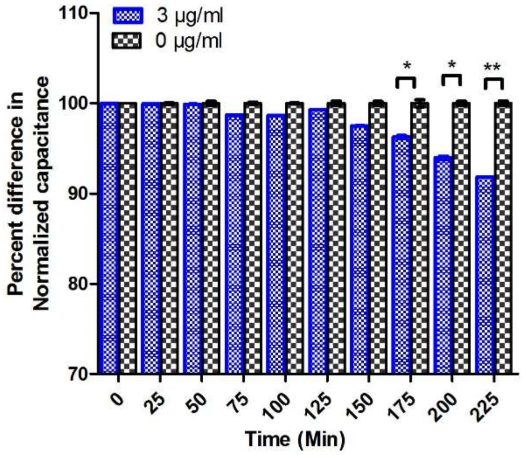

(**b**)

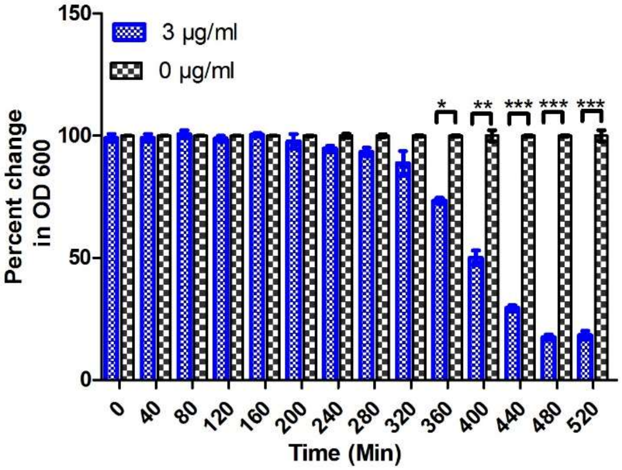

(**c**)

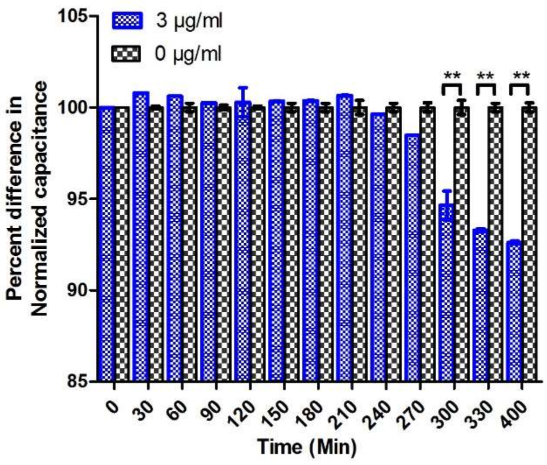

(**d**)

Supplementary Figure 4. Graphs are representing AST in the artificial urine samples using 3.0 μg/mL ampicillin: (**a**) The percentage change in the OD600 between control and antibiotic-treated 10^6 CFU/mL *E. coli* samples, (**b**) The percentage change in the normalized capacitance between control and antibiotic-treated 10^6 CFU/mL *E. coli* samples, (**c**) The percentage change in OD600 between control and antibiotic-treated in 10^4 CFU/mL *E. coli* samples; (**d**) The percentage change in the normalized capacitance between control and antibiotic-treated 10^4 CFU/mL *E. coli* samples. Error bars represent the standard deviation from the mean values of three experiments (significance indicators: *$p < 0.05$, ** $p < 0.01$, and *** $p < 0.001$).
